# THE EFFECT OF DUAL-TASKS ON WALKING SPEED IN HEALTHY OLDER ADULTS: A SYSTEMATIC REVIEW AND META-ANALYSIS

**DOI:** 10.1093/geroni/igad104.2137

**Published:** 2023-12-21

**Authors:** Hyeon Jung Heselton, Adam Rosen, Julie Blaskewicz Boron

**Affiliations:** University of Nebraska Omaha, Omaha, Nebraska, United States; University of Nebraska at Omaha, Omaha, Nebraska, United States; UNO, Omaha, Nebraska, United States

## Abstract

Normative age-related changes occur in both walking and cognitive performance; non-normative changes could lead to decreased mobility, loss of autonomy in daily life, and increased fall risk. This systematic review and meta-analysis aimed to assess the effects of completing a dual-task on overground walking speed in healthy older adults. Database searches were carried out in three electronic databases (PubMed, PsycINFO, ProQuest) from inception through October 2022 to find studies that assessed healthy older adults who completed a walking test and a walking task combined with a neurocognitive assessment (dual-task). Methodological quality and risk of bias was assessed via the Strengthening the Reporting of Observational Studies in Epidemiology (STROBE). Cohen’s d effect size with a 95% confidence intervals, Cochrane’s Q, I2, and Fail-safe N statistics were calculated between the single- and dual-task walking speeds. The initial search yielded 459 articles; six were included in the final review after completing all screening procedures. Included studies had relatively high STROBE scores (19.97±0.82). Eight individual effects across the six articles (total sample size=5627) were calculated. Walking speed significantly decreased in dual-task conditions with cognitive tasks (walking speed Δ=1.03; 95%CI 0.65 to 1.40; p<.001); individual effects were higher during high load cognitive tasks (i.e., serial 7’s subtraction, TMT part B). Dual-tasking with a cognitive component clearly affected walking speed in aging adults. Studying different age groups will help create a model of the aging process to understand when changes begin, as well as the rate of change; this could be useful for earlier fall-prevention interventions.

